# The time-resolved hard X-ray diffraction endstation KMC-3 XPP at BESSY II

**DOI:** 10.1107/S1600577521002484

**Published:** 2021-03-19

**Authors:** Matthias Rössle, Wolfram Leitenberger, Matthias Reinhardt, Azize Koç, Jan Pudell, Christelle Kwamen, Matias Bargheer

**Affiliations:** a Helmholtz-Zentrum Berlin für Materialien und Energie, Wilhelm-Conrad-Röntgen Campus, BESSY II, Albert-Einstein-Strasse 15, 12489 Berlin, Germany; b Institut für Physik and Astronomie, Universität Potsdam, Karl-Liebknecht-Strasse 24–25, 14476 Potsdam, Germany

**Keywords:** beamline instrumentation, time-resolved X-ray diffraction, optical excitation, thermal transport, ferroelectric switching

## Abstract

The time-resolved hard X-ray diffraction endstation KMC-3 XPP for optical pump/X-ray probe experiments at the electron storage ring BESSY II is dedicated to investigating the structural response of thin film samples and heterostructures after their excitation with ultrashort laser pulses and/or electric field pulses. It enables experiments with access to symmetric and asymmetric Bragg reflections via a four-circle diffractometer and it is possible to keep the sample in high vacuum and vary the sample temperature between ∼15 K and 350 K. The femtosecond laser system permanently installed at the beamline allows for optical excitation of the sample at 1028 nm. A non-linear optical setup enables the sample excitation also at 514 nm and 343 nm. A time-resolution of 17 ps is achieved with the ‘low-α’ operation mode of the storage ring and an electronic variation of the delay between optical pump and hard X-ray probe pulse conveniently accesses picosecond to microsecond timescales. Direct time-resolved detection of the diffracted hard X-ray synchrotron pulses use a gated area pixel detector or a fast point detector in single photon counting mode. The range of experiments that are reliably conducted at the endstation and that detect structural dynamics of samples excited by laser pulses or electric fields are presented.

## Abstract 2

The KMC-3 XPP endstation of the synchrotron BESSY II is dedicated to time-resolved studies of structural dynamics of matter upon optical and/or electrical excitation using hard X-ray diffraction with an accessible time range from 17 ps to several microseconds.

## 1. Introduction   

KMC-3 XPP is a hard X-ray synchrotron radiation beamline installed downstream of a dipole bending magnet source. It emits a broad usable X-ray energy spectrum between 2.5 and 20 keV that can be used for X-ray diffraction experiments or X-ray absorption spectroscopy (Schuck & Zisak, 2020 ▸). This article focuses on the capabilities of the ultrafast time-resolved X-ray diffraction at the X-ray pump–probe (XPP) endstation. Thin films, heterostructures, or bulk single-crystalline samples are optically or electrically excited and their temporal structural response is investigated using time-resolved X-ray diffraction with the time resolution given by the X-ray pulse duration of the synchrotron BESSY II. Time-resolved hard X-ray diffraction is a technique to directly study the structural response of matter in the time domain. In the following, we highlight the potential of the XPP endstation and describe the significant improvements compared with previous installations (Navirian *et al.*, 2012 ▸), especially in view of laser excitation and electrical field studies.

In pump–probe experiments the lattice response of the sample is probed by an X-ray pulse as a function of the time delay, τ, after sample excitation. Studies at the previous installation at the same bending magnet include the excitation and damping of acoustic phonon modes (Shayduk *et al.*, 2013 ▸) and their interaction with ferroelastic domain walls in SrTiO_3_ (Maerten *et al.*, 2018 ▸), heat conduction in heterostructures of oxide materials (Shayduk *et al.*, 2011 ▸; Navirian *et al.*, 2014 ▸), and through nanolayered rare earth samples (Koc *et al.*, 2017*a* ▸,*b* ▸). The technical challenges associated with the limited sample area excited by the pump pulse (Reinhardt *et al.*, 2016 ▸) have been solved by implementing a laser system that supports higher pulse energies and is thus able to excite larger pump areas with the same laser fluence. The gating time of the detectors used in these pump–probe experiments is much slower than the time-resolution given by the pulse duration but fast enough to single out one electron bunch to which the laser pulses are synchronized.

Pump–probe experiments with hard X-rays derived from the synchrotron radiation are conducted at various electron storage rings; however, each setup has a dedicated sample infrastructure and the parameters of the optical excitation pulses vary with respect to the pump wavelengths, pulse duration, and repetition rate of the experiments (Inoue *et al.*, 2001 ▸; LeGrand *et al.*, 2002 ▸; Beaud *et al.*, 2007 ▸; Ingold *et al.*, 2007 ▸; Nozawa *et al.*, 2007 ▸; Cammarata *et al.*, 2009 ▸; Dufresne *et al.*, 2011 ▸; Graber *et al.*, 2011 ▸; Ibrahimkutty *et al.*, 2011 ▸; Walko *et al.*, 2016 ▸; Bachiller-Perea *et al.*, 2020 ▸). Some of the endstations offer the possibility to use a white (Cammarata *et al.*, 2009 ▸; Graber *et al.*, 2011 ▸) or pink probe beam (Inoue *et al.*, 2001 ▸; Nozawa *et al.*, 2007 ▸), respectively, in contrast to the monochromatic probe beam at KMC-3 XPP.

A second class of time-resolved experiments that are regularly conducted at the KMC-3 XPP explicitly uses the time resolution of the detector as opposed to the time-delay between an optical pump and X-ray probe pulse. Recently, we have investigated the temporal response of ferroelectric materials such as Pb(Zr_*x*_,Ti_1–*x*_)O_3_ during the polarization reversal that is induced by electrical field pulses (Kwamen *et al.*, 2017 ▸, 2019 ▸). The surface acoustic waves in graphene and other materials were studied via piezoelectric excitation of these waves in lanthanum–gallium silicates (Roshchupkin *et al.*, 2013 ▸, 2014 ▸). Magnetic fields and electric fields as well as photostriction may modify the lattice constant of magnetoelectric materials such as BiFeO_3_ (Iurchuk *et al.*, 2016 ▸). In these experiments the timing of the electrical excitation pulse and the detector is determined electronically, either by single photon counting modules or gated pixel detectors. Technically similar experiments on ferroelectric or piezoelectric activity have been conducted at other synchrotron radiation facilities (Lee *et al.*, 2001 ▸; Do *et al.*, 2008 ▸; Pramanick *et al.*, 2009 ▸; Gorfman *et al.*, 2015 ▸; Wallace *et al.*, 2015 ▸; Davydok *et al.*, 2016 ▸; Ehara *et al.*, 2017 ▸); however, the sample environment at the KMC-3 XPP permits simultaneous characterization of the electric currents in the device for a complete operando characterization of structural and electric properties.

The paper is structured as follows: Section 2.1 introduces the layout of the KMC-3 beamline and in Section 2.2 the XPP endstation is described. We explain the time-resolved experiments with laser excitation in Section 2.3 and electrical field excitation in Section 2.4, respectively. The available detectors and routinely used detection schemes are introduced in Section 2.5. Section 3 presents experiments that demonstrate the possibilities at the XPP endstation. We discuss experiments on the ultrafast strain generated in SrRuO_3_ in Section 3.1.1. The special low-α operation mode of BESSY II is used for experiments with improved time-resolution (Abo-Bakr *et al.*, 2002 ▸, 2003 ▸). The temporally shortened X-ray single bunch allows us to measure the ultrafast SrRuO_3_ expansion presented in Section 3.1.2 as well as the dynamics in rare earth metal thin films like Dy in Section 3.1.3. We explore the other end of the available timing range in the nano- to micro-second range in experiments on thermal transport in nanometric functionalized Au nanotriangles in Section 3.2. Section 3.3 reports on the electrical excitation and polarization reversal of ferroelectric thin films. We conclude with a summary and outline future excitation schemes available at the beamline in the near future in Section 4.

## 2. Beamline and endstation   

### 2.1. Layout of the KMC-3 beamline   

**Figure 1:**
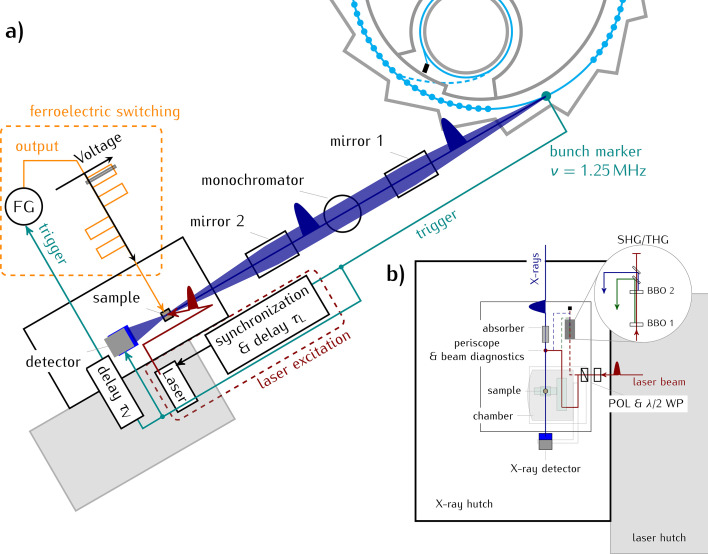
Layout of the KMC-3 beamline and the XPP endstation at BESSY II. (*a*) The separated single bunch (indicated by the enlarged dark cyan circle within the ion clearing gap) circulates around the BESSY II storage ring and emits X-ray pulses sketched in dark blue. The divergence of the emitted X-ray beam is parallelized by mirror 1, the wavelength is selected by a Si (111) double-crystal monochromator, and the X-ray beam is finally focused onto the sample by mirror 2. The X-rays diffracted from the sample are counted by a gated pixel area detector or scintillator with photomultiplier. The bunch marker signal at 1.25 MHz from the storage ring trigger is used to synchronize the pump laser pulses (shown in dark red). A function generator (FG) triggered by the laser provides a voltage pulse sequences for sample excitation (indicated in orange), and the gate pulse for the detector synchronized to the synchrotron frequency, ν. An electronic delay unit allows shifting of the laser timing τ_L_ with respect to ν; the same is possible for the delay τ_V_ of the voltage sequence for ferroelectric switching studies as indicated by the orange voltage pattern. (*b*) Top view of the experimental hutch for the time-resolved diffraction experiments. The inset shows the alternative generation of second and third harmonic laser light. ‘POL & λ/2 WP’ indicates the polarizer motorized half-wave plate combination that changes the incident laser pump power on the sample.

**Figure 2:**
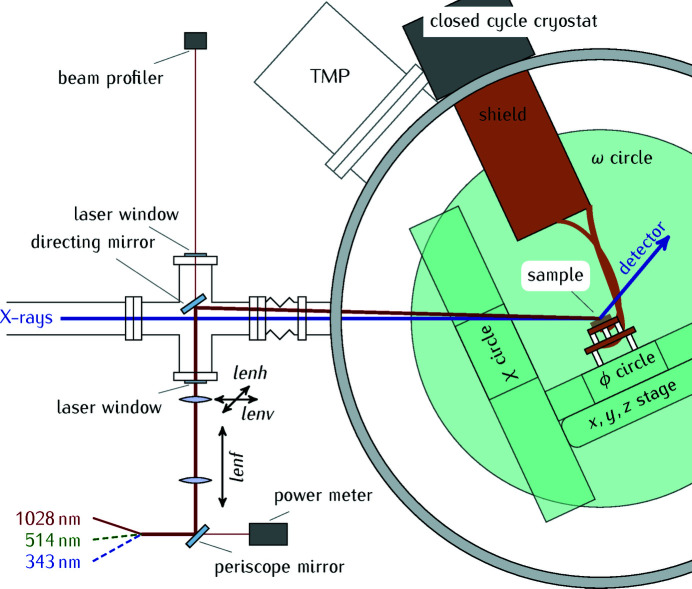
Side view and laser beam path in the diffraction chamber. The sample is mounted inside a vacuum chamber on a cryogenically cooled sample holder attached to the three-circle goniometer; the detector is mounted on the 2θ arm moving outside of the evacuated chamber (not shown here). The pump laser is coupled into the chamber through a quartz window attached to the evacuated beam tube. The last lens is motorized and used for the optimization of the spatial overlap of X-ray and laser spot on the sample (*lenv*, *lenh*); the laser spot size on the sample is adjusted by the movement of the second lens (*lenf*). A power meter and beam profiler allow to monitor the laser beam parameters. The laser and the X-ray beams have an angular offset of ∼2°.

The KMC-3 synchrotron radiation beamline is located at the dipole bending magnet 13.2 of the electron storage ring BESSY II of the Helmholtz-Zentrum Berlin. In Fig. 1 ▸(*a*) we show schematically the layout of the storage ring with the electron trajectory drawn in cyan and the emitted X-ray beam shown in blue. The emitted divergent X-ray beam is collimated with a parabolic mirror (Rh- and Pt-coated Si substrate, Bruker), monochromated by a Si (111) double-crystal monochromator (FMB Oxford), and focused onto the sample by a second parabolic mirror (Zizak & Gaal, 2017 ▸). The typical size of the X-ray focus at the sample position is horizontally 150 µm and vertically 400 µm and can additionally be shaped by aperture slits installed 80 cm before the sample. Typical values of the available X-ray flux at the sample position are given for different energies in Table 1 ▸. If necessary, the intensity of the X-ray beam can be attenuated by the insertion of Al foil filters before the X-ray beam impinges on the sample. The sample is mounted in a diffraction chamber as sketched in Fig. 2 ▸. The diffracted photons are measured using different X-ray detectors using time-resolving detection schemes explained in Section 2.5.

### 2.2. Sample environment and diffraction geometry   

The XPP endstation is set up in two adjacent experimental hutches as indicated in Fig. 1 ▸(*b*). In the X-ray hutch the diffraction experiments are performed; the laser hutch contains the laser system with the laser timing and diagnostics and additional space for other optical setups, for example for a non-collinear optical parametric amplifier (NOPA) that will soon be commissioned.

The sample can be kept in a high vacuum environment during the X-ray diffraction experiments. In Fig. 2 ▸ we show schematically the sample environment. The X-ray beam is guided through an evacuated tube that is directly attached to the stainless steel diffraction chamber with a base pressure of <10^−6^ mbar in which the in-vacuum three-circle goniometer (Huber Diffraktions­technik) is mounted. The 2θ arm that holds the detectors is installed outside of the vacuum chamber. The chamber is evacuated by a scroll pump (Edwards Vacuum) and a turbo molecular pump (Leybold TurboVac Mag), the latter being directly attached to the experimental chamber. Sample temperatures between ∼15 K and 350 K are achieved using a cryocooler with closed helium cycle (Sumitomo). Copper braids are attached to the first and second stage of the cryocooler and cool the thermal insulation shield to 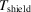 ≃ 70 K and the sample holder to 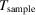 ≃ 15 K, respectively. The sample holder is thermally insulated from the plate carrying the shield by means of three ceramic spacers. In addition, the mounting plate attached to the goniometer is also thermally decoupled from the plate carrying the shield by ceramic spacers. A heater cartridge is used to stabilize and control the sample temperatures between ∼15 K and 350 K with a LakeShore336 temperature controller that uses the input of two silicon temperature diodes (LakeShore) attached to the sample holder and the mounting plate. In experiments with laser excitation, the stationary increase of the film temperature strongly depends on the optical absorption of the sample, the excitation fluence, and the heat conduction through the sample substrate. Typically, we observe a static heating due to laser illumination on the order of ∼80 K for metallic samples with moderate excitation fluences of *F* = 5–10 mJ cm^−2^ at a repetition rate of 104 kHz. For weakly absorbing samples, the temperature increase is dramatically reduced to only a few K. Usually we fix the samples with silver paint on copper slabs that are directly screwed to the cold part of the sample holder, which allows for quick exchange of samples.

The four-circle goniometer allows rotating the sample by the Eulerian angles ϕ, χ, ω, which correspond to rotations about the sample surface normal, about the X-ray beam direction, and about the horizontal direction perpendicular to the X-ray beam, respectively. Thus, it is possible to access Bragg reflections with different in-plane and out-of-plane components. It is possible to orient the sample such that the X-ray footprint on the sample remains constant when access­ing different asymmetric Bragg reflections. The sample position is adjusted with the help of three (*x*, *y*, *z*) linear stages to the center of the diffractometer, which coincides with the X-ray beam position. The detector arm, that is the 2θ circle, moves the detectors along a vertical circle outside the vacuum chamber. This geometry maximizes the scattering efficiency of the *p*-polarized X-rays. The diffracted X-ray photons leave the vacuum chamber through a differentially pumped rectangular Kapton window (DuPont) covering the angular range −2° < 2θ < 120°. All motions of the sample and the detector are controlled by the experimental control software *SPEC* (Certified Scientific Software), which is also used to orient the sample in reciprocal space.

### 2.3. Time-resolved optical pump/X-ray probe experiments   

Now we describe the experimental setup for the time-resolved optical pump/X-ray probe experiments. We use a commercial amplifier laser system (Pharos, LightConversion) for the optical excitation. It emits the fundamental wavelength of λ = 1028 nm and offers a maximum pulse energy of ∼400 µJ at laser repetition rates ν_L_ < 50 kHz. When synchronized to the storage ring, the repetition rate of the laser ν_L_ can be varied between 1 < ν_L_ ≤ 625 kHz and the pulse duration can be varied using the built-in compressor between ∼600 fs and 35 ps (FWHM value). The maximum average output power of the amplifier is 20 W; however, at lower repetition rates the maximum pulse energy limits the total output power. Typically the laser is operated at the output power of 15 W and at ν_L_ = 104 kHz, that is, every 12th round-trip of the camshaft electron bunch is used. Higher laser repetition rates are advantageous if the structural response is small: this typically is the case for low fluence excitation conditions or for weakly absorbing samples where also the heat load is significantly reduced. Increasing the repetition rate of the laser by a factor of up to six to the maximum of 625 kHz increases the available X-ray flux by the same factor. At this maximum repetition rate the laser pumps every second X-ray pulse emitted from the camshaft bunch.

To stabilize the laser repetition rate of the laser system and synchronize it to the X-ray pulse pattern, the repetition rate of the laser oscillator is synchronized to the radiofrequency ν_RF_ = 500 MHz of the synchrotron with a Syncro RRE unit (Menlo Systems) that drives two piezo actuators (fine and coarse) in the oscillator cavity. The relative delay between laser pulse and X-ray pulse timing is adjusted with a Menlo DDS120 direct digitizer unit that detunes the frequency or the phase of the laser oscillator frequency relative to the radiofrequency (RF) synchrotron frequency. This allows us to electronically realize delays τ_L_ of up to several µs given by 1/ν_L_, the inverse of the laser repetition rate. The synchronization uncertainty between laser and between the RF frequency reference signal that is delivered to the beamline and the laser timing derived from the oscillator frequency is better than 500 fs as inferred from the power spectral density output signal of the synchronization device. The laser timing is routinely monitored with a photodiode (Hamamatsu) in single photon counting mode together with a PicoHarp 300 single photon counting module. A Python program analyzes the histogram of the PicoHarp module to verify the laser–X-ray timing while changing the delay τ_L_ during extended delay scans. The timing offset is calibrated using a fast responding sample, for example a SrRuO_3_ thin film, as will be discussed in Section 3.1.

In order to optimize the spatial overlap between laser and X-ray spot, the last focusing lens is motorized. With the help of a camera the laser is roughly aligned to the sample and the X-ray position. Slight movements of the motorized optical focusing lens with respect to the laser beam vertically (*lenv*) and horizontally (*lenh*) relative to the X-ray direction results in a sufficiently large movement of the laser spot on the sample, see Fig. 2 ▸. Scanning both dimensions yields the optimum laser–X-ray overlap by maximizing a certain feature of the diffraction pattern, for example the shift of a Bragg peak shoulder (Reinhardt *et al.*, 2016 ▸). We check and optimize the spatial overlap of pump and probe beams on a reference sample that consists of a 157 nm-thick metallic SrRuO_3_ layer grown by pulsed laser deposition on a (001) SrTiO_3_ substrate, following the procedure presented by Reinhardt *et al.* (2016 ▸). The temporal overlap is also checked with the same SrRuO_3_ reference sample, which has a quasi-instantaneous response of ∼10 ps that is much faster than the temporal resolution of the X-ray pulses in BESSY’s hybrid mode as we will show in Section 3.1.1 and discuss in Section 3.1.2 in more detail.

The laser power is monitored using a powermeter (Thorlabs S120VC), which is positioned behind the second last directing mirror, and a beam profiler after the last steering mirror, see Fig. 2 ▸. A motorized half-wave-plate–polarizer combination (Thorlabs) is used to adjust the laser power remotely with a LabView program. This program also reads out the powermeter. With a second mobile powermeter temporarily placed in the diffraction chamber it is possible to calibrate the permanently installed powermeter–half-wave-plate–polarizer combination such that the power or the fluence can be adjusted remotely during the experiment even when the chamber is closed. The latter is possible because the beam profiler is installed at the same distance from the last steering mirror as the sample, which ensures that the beam diameter at the sample position and on the beam profiler is the same. This allows for monitoring the beam diameter and position *in situ* even with the evacuated sample chamber and a cooled sample. The laser spot size can be adjusted by a movement of the first focusing lens (*lenf*) along the optical axis between 0.2 < *d* < 1 mm spot diameter, which changes the excitation fluence, *F*, without changing the average heat load on the sample. This automated setup allows for compensating changes of the pump spot size and consequently of the pump fluence on the sample in different diffraction geometries.

Using a non-linear optical setup installed in the X-ray hutch, samples can be excited at different wavelengths according to their optical properties: in particular, green 514 nm light, which is generated by frequency-doubling in a β-bariumborate (BBO) crystal, and ultraviolet 343 nm light, which is produced in a second BBO where the fundamental wavelength and the second harmonic light undergo sum-frequency generation (Homann *et al.*, 2008 ▸), are readily available. This setup reaches up to almost 50% conversion efficiency for 514 nm light (typically tuned to ∼30%) and 10% efficiency for 343 nm light.

### 2.4. Time-resolved piezo-electric or ferroelectric response   

**Figure 3:**
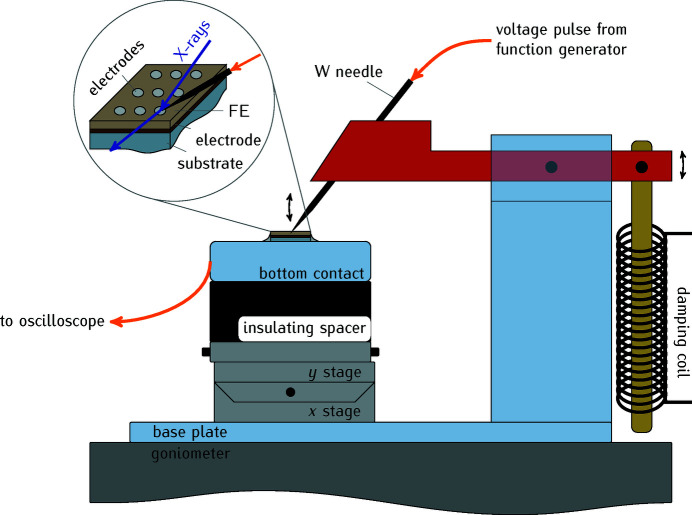
Setup for the application of voltage pulse sequences to FE test devices. The sample is glued on a conducting metal holder that is connected to an oscilloscope for the measurement of the FE switching current. A W needle establishes the electrical contact to a metallic top electrode (see also the inset). In order to change between different top electrodes, the needle can remotely lifted up and the sample moved relatively to the needle with the *x*, *y* stages. The plastic needle holder movement is remotely operated and its lowering motion is damped with an induction coil.

For time-resolved experiments on piezoelectric or ferroelectric structure dynamics it is necessary to connect the sample to an electrical power supply without disturbing the X-ray detection. To this end, we developed a special sample holder for the time-resolved operando investigation of thin film crystal structure during the polarization reversal. Fig. 3 ▸ shows a sketch of the sample holder. It permits time-resolved X-ray diffraction measurements during the polarization reversal of ferroelectrics (FE) while simultaneously recording the switching current in the circuit (Wooldridge *et al.*, 2012 ▸; Ryding *et al.*, 2013 ▸; Fancher *et al.*, 2017 ▸; Kwamen *et al.*, 2017 ▸).

The FE sample holder replaces the low-temperature sample holder on the goniometer in the vacuum chamber presented in Fig. 1 ▸(*b*) and enables automated electrical contacting of small test devices. We preferably use laterally structured samples with circular or hexagonally shaped electrodes with diameters between 200 and 400 µm. These are usually thermally evaporated or sputtered onto the device surface and shaped using a shadow mask. Different metals like Al, Cu, or Pt are reasonable choices, depending on the switching properties of the FE (Pintilie *et al.*, 2008 ▸). Using a tungsten needle with a tip radius of either 1 or 5 µm (EPP, Germany), we can selectively apply the gate voltage to one single contact. The samples are glued on a metallic carrier with silver paint and, thus, the bottom electrode of the device is connected to the measurement circuit either via excess silver paint that connects the bottom electrode from the sample side(s), or through a conducting substrate to the oscilloscope. With this setup we are limited to switching times of 10 ns. For faster electronic dynamics the impedance matching has to be carefully considered (Grigoriev *et al.*, 2011 ▸).

The sample can be moved with a small *x*, *y* stage (Owis KTM40) relative to the needle for an automated local electrical contacting. For the selection of an electrical contact, the needle can be lifted up and lowered as indicated by the arrows in Fig. 3 ▸. This is important because the movement of the sample with the lowered needle may scratch the sample surface and bend the needle tip. The lowering motion of the needle is damped by an induction coil that is powered from an external control box and places the needle carefully on the sensitive sample surface. In essence, the different contacts can be moved into the X-ray spot and contacted with the needle. The electrical connections to the sample holder are realized with BNC cables, which are connected outside of the experimental hutch to the function generator (Keithley 3390) that supplies the voltage pulse sequence and to an oscilloscope (Agilent DSO9404A) that records the switching current of the ferroelectric material. Low switching currents can be amplified with a current amplifier (Femto DLPCA series).

For the time-resolved measurements with applied electric fields, the function generator applies a periodic voltage pulse sequence to the sample with a certain repetition rate. This signal also triggers the time correlated single photon counting (TCSPC) of the X-rays (PicoHarp). A typical example of a PUND sequence (Setter *et al.*, 2006 ▸) consisting of Positive Up and Negative Down pulses (Traynor *et al.*, 1997 ▸; Fina *et al.*, 2011 ▸) is sketched in orange in the inset of Fig. 1 ▸. Alternatively, other arbitrarily shaped voltages sequences can be applied, for example triangular voltage ramps.

### 2.5. Detection schemes for time-resolved experiments with different excitation conditions   

We use for the time-resolved X-ray diffraction experiments at KMC-3 XPP different excitation and adopted detection schemes. In this section, we present and discuss these schemes and their respective advantages. In general, for most of the time-resolved X-ray diffraction experiments, we make use of the particular filling patterns of the BESSY II storage ring (Holldack *et al.*, 2007 ▸; Müller *et al.*, 2016 ▸). In Fig. 1 ▸(*a*), we sketched the ‘usual’ filling pattern of the storage ring, the so-called hybrid mode, where 302 out of total 400 electron buckets are filled (indicated by the cyan filled circles). An additional bunch (drawn in green), the so-called camshaft or single bunch, is situated within a 200 ns long ion clearing gap. The average ring current is 300 mA and the camshaft bunch holds an equivalent current of ∼4 mA. During normal operation, the current is refilled approximately every 90 s and the ring current is kept constant. The round trip time of the electron bunches in the storage of BESSY II is ∼800 ns. A modified operation mode of BESSY II with only one single bunch and a current of 15 mA in the storage ring is available for two weeks per year. In both modes, the time-resolution is limited by the electron bunch length to approximately 80 ps. For time-resolved experiments with improved time-resolution the so-called ‘low-α’ operation mode can be used. In this special operation mode with special settings of the storage ring electron optics, 336 buckets are filled and the average ring current is 100 mA with 0.3 mA current stored in the camshaft bunch. However, the low-α mode is operated in decay mode, that is, the current and hence the number of photons reduces with time. Every 8 h at 7am, 5pm, and 11pm, the storage ring is refilled.

For the time-resolved measurements a Dectris Pilatus 100k pixel area detector (Henrich *et al.*, 2009 ▸) and a home-built fast scintillation point detector are simultaneously mounted outside of the vacuum chamber on the 2θ arm. Both detectors are operated in single photon counting mode during the time-resolved experiments. A scintillation detector (X2000 Cyberstar) with large detector opening can be additionally used for measurements without time-resolution. The home-built scintillation detector consists of a plastic scintillator with decay time <1 ns (Scionix), which converts X-ray photons into optical photons with an efficiency ∼10 photons keV^−1^. The photons are detected by a photomultiplier tube (Hamamatsu H7844) connected to a current amplifier (Femto LCA series) or a photomultiplier detector module (PicoQuant PMA) equipped with the same scintillator material. The amplified signal is sent to another single photon counting module PicoHarp 300 (PicoQuant). This allows for example directly measuring the filling pattern of BESSY II with sub-nano­second resolution as demonstrated by Shayduk *et al.* (2011 ▸).

Correct gating of the detectors ensures that only the X-ray pulse generated from the single electron bunch is detected. Therefore the laser and the detectors are synchronized to the arrival time of the X-ray pulses that is derived from the synchrotron radiofrequency 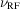 ≃ 500 MHz, which is also fed into the laser synchronization unit (see Section 2.3). A delay generator (Stanford Research Systems DG645) generates the detector gate signal (Navirian *et al.*, 2012 ▸), which enables the area pixel detector at the repetition rate of the laser only during the arrival of the camshaft X-ray pulses to which the laser-excitation is synchronized (Ejdrup *et al.*, 2009 ▸). Using a gated detector allows us to work without complex mechanical devices like high-speed choppers (Cammarata *et al.*, 2009 ▸; Plogmaker *et al.*, 2012 ▸; Förster *et al.*, 2015 ▸) or excitation of the electron bunches either with a laser (Beaud *et al.*, 2007 ▸) or the local manipulation of the electron orbit (Holldack *et al.*, 2014 ▸). Since the detector readout time is much slower than the repetition rate of the experiment (0.2 kHz versus 100 kHz), the area detector is operated in the so-called ‘external enable mode’ with the repetition rate of the laser. The resulting image of the accumulated snapshots is read out after the exposure. The number of images is given by the user-defined integration time and the repetition rate. Alternatively, we use the point-detector, which records the arrival time of X-rays with respect of the single bunch using the single photon counting module.

The accompanying structural information is either measured in the form of full reciprocal space maps (RSMs) at defined time delays τ_L_ or τ_V_ by triggering the 100 ns gate of the area detector, or by measuring θ–2θ scans with the point detector. The fast scintillator with photomultiplier detector is directly read out by the PicoHarp module. The time resolution is limited by the decay time of the scintillator to approximately 2 ns. This acquisition mode directly uses the 16-bit analog-to-digital converter (ADC) of the PicoHarp to simultaneously acquire 2^16^ time channels with a temporal gate width of 512 ps, which amounts to a simultaneous measurement time window up to 33 µs after a trigger pulse has started the measurement (Shayduk *et al.*, 2011 ▸; Navirian *et al.*, 2012 ▸). The diffraction curves for every time channel are subsequently extracted using a Matlab or Python script as described in Section 3.3.

It is rather time-consuming to record full RSMs with the point detector since both ω and 2θ would have to be scanned. In some cases it can nonetheless be beneficial to use the point detector even though it measures only a cut of the reciprocal space during the scan because the X-ray diffraction on the few nanosecond to microsecond timescale can be recorded simultaneously during the RSM scan. This is possible because the single photon counting module time-stamps all the detected X-rays. For an illustrative example see Section 3.3 where we demonstrate that it is sufficient to use the point detector for a θ–2θ scan (more precisely an ω–2θ scan with ω = θ), for example to measure the distance *d* of lattice planes according to Bragg’s law. To assess the mosaicity of a sample, ω-scans with the detector at a fixed 2θ angle are used.

To construct RSMs of the samples around a certain reciprocal lattice vector **G**, we orient **G** such that together with the incoming X-rays with wavevector **k** it spans a vertical diffraction plane that permits the Bragg-diffracted X-rays to exit the vacuum chamber through the Kapton window. Then we vary the angle ω between **k** and the lattice planes perpendicular **G** and detect the scattered X-rays in the vicinity of the Bragg reflection, *i.e.* at a scattering angle of approximately 2θ. The area detector simultaneously measures scattered X-rays **k**′ = **k** + **G** + Δ**q** which may deviate from Laue’s scattering condition by wavevectors Δ**q** given by the mosaicity and coherence length of the diffracting sample. The intensity map is transformed into the reciprocal space of the sample by a Python program based on the Python library xrayutilities (Kriegner *et al.*, 2013 ▸) to construct the RSM *I*(*q*_*x*_, *q*_*y*_, *q*_*z*_). Typically, we analyze the RSM by integrating out the *q*_*x*_ or *q*_*y*_ direction perpendicular to the scattering plane. If the area detector is not moved during the ω variation in an RSM scan, the resolution of the RSM in the *q*_*x*_ direction is optimized whereas along *q*_*z*_ it is optimized by maintaining the Bragg condition 2θ = 2ω, which ensures that the symmetrically diffracted X-rays are always detected by the same pixels of the detector during the scan.

## 3. Typical experiments conducted at KMC-3 XPP   

In the following section we present and discuss experiments that are routinely conducted at KMC-3 XPP. We put the emphasis on the illustration of results for potential users that are not working with time-resolved diffraction experiments on a daily basis. This experimental part of the paper is divided into separate sections dealing with optical excitation of heterostructures shown in Section 3.1, the optical excitation of mosaic samples in the form of gold nanotriangles in Section 3.2, and the *in operando* investigation of ferroelectrics presented in Section 3.3. We emphasize that the time scale of the experiments at KMC-3 XPP cover the time range from a few tens of picoseconds for the low-α experiments with optical excitation over slow dynamics of several hundred nano­seconds seen in Dy heterostructures up to the microsecond time scale observed in the ferroelectric switching studies.

### 3.1. Transient strain generation by optical excitation   

We start with typical pump–probe experiments by discussing the transient response of the SrRuO_3_ layer that we routinely use for checking the timing of the optical pump/X-ray probe setup. Such films exhibit a rhombohedral structure that is usually described by pseudo-cubic Miller indices (Vailionis *et al.*, 2011 ▸). The properties of SrRuO_3_ thin films have been extensively studied and summarized in the review article by Koster *et al.* (2012 ▸). We have performed several time-resolved investigations of the structural response of SrRuO_3_ (Korff Schmising *et al.*, 2006 ▸; Navirian *et al.*, 2010 ▸; Gaal *et al.*, 2012 ▸; Herzog *et al.*, 2012 ▸; Bojahr *et al.*, 2015 ▸), which indicate that this is a suitable ‘reference’ sample because of its excellent crystalline quality and high damage threshold. In the following, we show and discuss experiments in hybrid mode of BESSY II and study the fast sample response using the low-α mode of BESSY II.

#### 3.1.1. Dynamics of a SrRuO_3_ thin film in BESSY’s hybrid operation mode   

**Figure 4:**
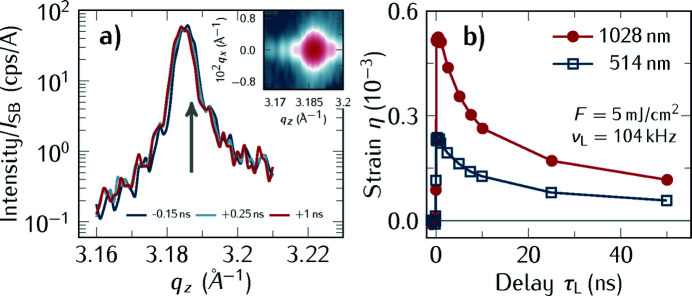
Time-resolved optical pump/X-ray probe experiments of a 157 nm-thick SrRuO_3_ layer on a SrTiO_3_ substrate at *T* = 300 K. (*a*) Integration of the reciprocal space maps along *q*_*x*_ showing the temporal evolution of the out-of-plane lattice vector *q*_*z*_ for the SrRuO_3_ 002 lattice vector after excitation with λ = 514 nm at the repetition rate ν = 104 kHz with fluence *F* = 5 mJ cm^−2^. The vertical gray arrow indicates the peak position without laser heating. The inset of (*a*) is the RSM of the measurement at τ_L_ = +140 ps for λ = 514 nm. (*b*) Strain η as function of the delay τ_L_ induced by the optical excitation of the SrRuO_3_ layer with ultrashort laser pulses with 1028 nm wavelength (red circles) and 514 nm (open blue squares).

In the following, we first discuss experiments performed in the hybrid operation mode of BESSY II using the gated Pilatus area detector. In Fig. 4 ▸(*b*) we show a time-resolved reciprocal space map of the 002 Bragg reflection of SrRuO_3_ before and after laser excitation with λ = 514 nm with the incident fluence *F* = 5 mJ cm^−2^ at the laser repetition rate ν_L_ = 104 kHz. Note that the intensity shown in the plots is normalized to the single bunch current. It is important to normalize the diffraction intensity to the single bunch current in order to correct for the decaying intensity of the synchrotron radiation between the subsequent refills of the electrons into the storage ring. These typical RSM scans take around 5 min per delay, hence recording the full transient shown in Fig. 4 ▸ took roughly 1.5 h per fluence.

Fig. 4 ▸(*a*) shows the time-resolved X-ray diffraction measurements of the 002 Bragg reflection of the SrRuO_3_ layer after excitation with λ = 514 nm. These curves originate from an integration of the RSM along *q*_*x*_. The gray arrow in Fig. 4 ▸(*a*) indicates the peak position of the SRO layer peak when the sample was not illuminated with laser light. From the shift of the curves at negative time delays with respect to this arrow, we estimate a static heating of the SRO layer at ν_L_ = 104 kHz of 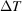 = 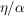 = 8 × 10^−4^/1.03 × 10^−5^ K^−1^ ≃ 78 K where η is the strain calculated from the Bragg peak shift and α is the linear thermal expansion coefficient taken from Yamanaka *et al.* (2004 ▸).

The analysis of the integrated RSMs in Fig. 4 ▸ at different delays τ_L_ yields the photo-induced strain η(τ_L_) = *c*(τ_L_)/*c*(τ_L_ < 0) − 1 derived from the change of the lattice constant, *c*. The transient strain is shown in Fig. 4 ▸(*b*) for the two excitation wavelengths 1028 nm and 514 nm, respectively. The difference of the generated strain is due to the different optical absorption coefficients of SrRuO_3_: the imaginary parts of the refractive index κ_1028 nm_ = 1.87 compared with κ_514 nm_ = 0.94 are determined by spectral ellipsometry. Both experiments were conducted with the same illumination spot size and incident laser fluence. We note the quasi-instantaneous strain increase after the excitation when the SrRuO_3_ layer expands. The time of maximum expansion is determined by the layer thickness and its sound velocity (Shayduk *et al.*, 2013 ▸; Schick *et al.*, 2014*b* ▸) and we expect τ_exp,max_ = 157 nm/(6.3 nm ps^−1^) = 25 ps, thus the observed increase of the strain is limited by the X-ray pulse duration of 80 ps in the hybrid mode of the storage ring. After the maximum expansion, the heat flows diffusively out of the SrRuO_3_ layer on a several tens of nanoseconds time scale.

#### 3.1.2. Resolving the SrRuO_3_ thin film expansion in low-**α** operation mode   

**Figure 5:**
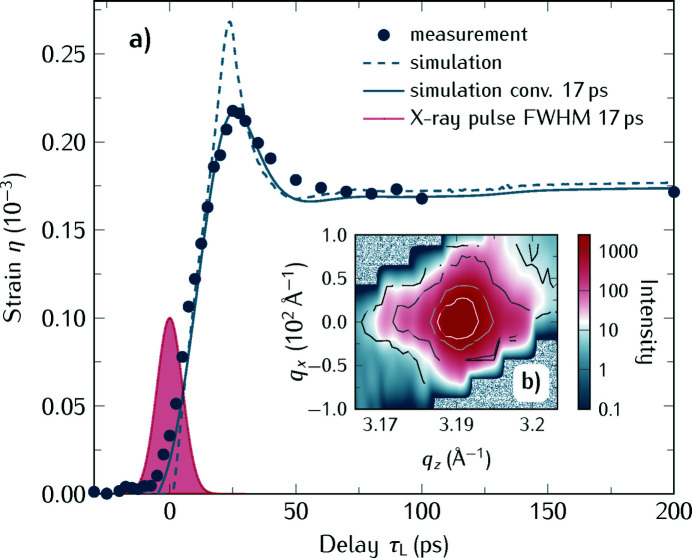
Time-resolved measurement of a SrRuO_3_ thin film after laser excitation in low-α operation mode of BESSY II. (*a*) Time-resolved strain η (filled blue symbols) in a 157 nm-thick layer after optical excitation at room temperature with 1028 nm using a fluence *F* = 2.5 mJ cm^−2^ as a function of delay τ_L_. A simulation of the transient strain using the udkm1Dsim toolbox (Schick *et al.*, 2014*a* ▸) is shown by the dashed blue line. Its convolution with a time-resolution of 17 ps (FWHM) is shown by the blue solid line. The pulse length of the X-ray pulses is indicated by the filled red Gaussian pulse at τ_L_ = 0. (*b*) RSM of the 002 Bragg reflection of SrRuO_3_. The color plot is a snapshot at τ_L_ = −40 ps before laser excitation and the thin lines on top are measured at τ_L_ = +40 ps.

In order to study the fast expansion of the SRO thin film within 25 ps after excitation we performed time-resolved measurements in the low-α mode of BESSY II on the same sample discussed before. We benefit from the improved time-resolution of the low-α operation due to the shortened electron bunches and can perform experiments with the best time-resolution available at the beamline. Even though the clearing gap around the single bunch in BESSY’s low-α mode is shortened to 128 ns, we are still able to measure with the gated area detector. The results of a measurement with *F* = 2.5 mJ cm^−2^ at room temperature with the laser wavelength λ = 1028 nm and repetition rate ν_L_ = 104 kHz is shown in Fig. 5 ▸(*a*) by the blue filled symbols.

We note that in this special case the delay τ_L_ between laser pulse and X-ray pulse is changed just by setting the phase at the DDS120 delay unit without checking the fast photodiode, that is, without feedback. The latter would lead to an intrinsic timing jitter of at least 4 ps as the time resolution of the PicoHarp is limited to 4 ps in its highest time-resolving mode. This modified procedure allows us to reliably set a delay step width of Δτ_L_ = 2 ps in low-α mode that results in a time resolution of ∼17 ps compared with 80 ps in hybride mode. We compare this result with a simulation of the laser-induced lattice dynamics of SrRuO_3_ on SrTiO_3_ obtained using the udkm1Dsim toolbox (Schick *et al.*, 2014*a* ▸) shown by the blue dashed line in Fig. 5 ▸(*a*). We obtain a good agreement between simulation and experiment after convolution of the simulation with a Gaussian function of 17 ps width (FWHM), which is given by the blue solid line. The time resolution is indicated by the filled red Gaussian profile with 17 ps FWHM at the onset of the strain transient, which is consistent with a 16 ps X-ray pulse length of the camshaft bunch in low-α ‘mode B’ operation. In Fig. 5 ▸(*b*) we show the RSMs for τ_L_ = ±40 ps. The experimental data in Fig. 5 ▸(*a*) were obtained from RSM scans at 42 time delay steps τ_L_, which were measured twice in 7 h, that is, one delay scan takes 3.5 h, which can easily be integrated into the 8 h injection schedule in low-α mode operation described before.

#### 3.1.3. Ultrafast lattice response of dysprosium   

In the following we exploit the possibility at KMC-3 XPP to change the delay τ_L_ between optical pump and X-ray probe pulse electronically over a wide temporal range, which enables us to reach delays from several picoseconds up to several hundred nanoseconds. This makes this particularly interesting for the investigation of heat transport on the nanoscale, which is an important research field for the development of new electronic devices. Rare earths metals like dysprosium are an interesting testbed of magnetic phenomena. The contributions of the spin system to the stress on the lattice show both contributions on a few picosecond timescale and on the nanosecond timescale (von Reppert *et al.*, 2016*a* ▸, 2020 ▸; Koc *et al.*, 2017*b* ▸). In these systems also the thermal transport by the phonons and electrons is influenced by the scattering of the heat conducting quasiparticles from spin excitations: Dy exhibits a ferromagnetic low-temperature phase that is superseded by an antiferromagnetic phase at 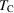 ≃ 90 K with a Neél temperature of 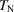 ≃ 179 K. In the antiferromagnetic phase a pronounced negative thermal expansion suggests a particularly strong spin–lattice interaction due to the magnetic properties of the Dy 4*f* electrons (Darnell & Moore, 1963 ▸).

**Figure 6:**
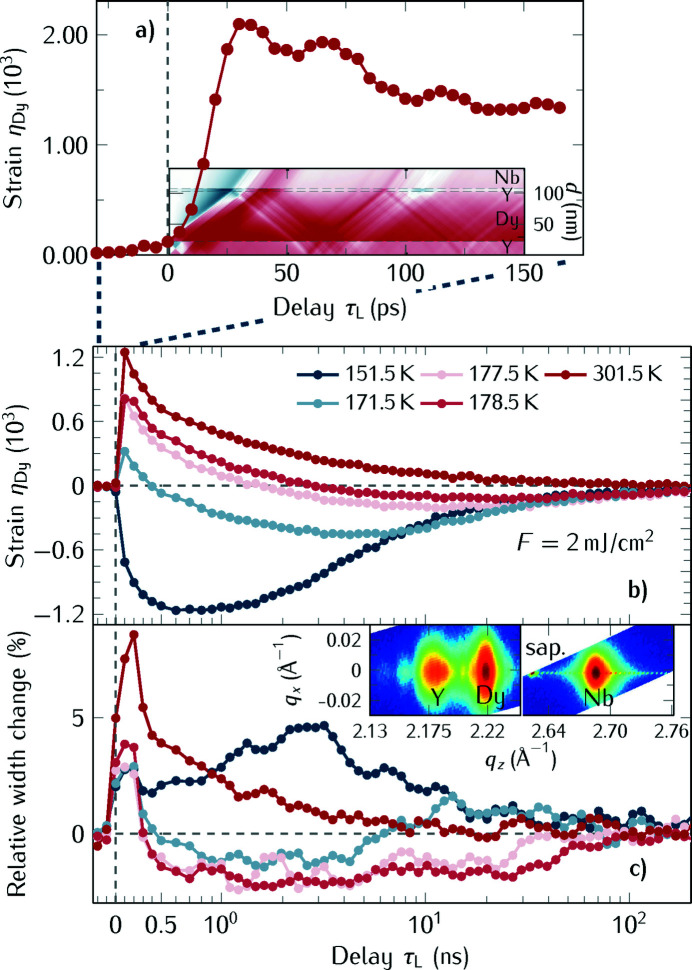
(*a*) Room temperature data of the strain measured in Dy in low-α mode reveal the acoustic oscillations of the Dy film. The inset shows a simulation of the strain in the heterostructure where the color represents the strain: blue encodes compressive strain, red expansive strain. Whenever the average strain in the Dy layer is large, the measurement shows a maximum. (*b*) Measured strain in the Dy layer as a function of the sample temperature. For temperatures below the Néel temperature a remarkable transient negative thermal expansion is observed. (*c*) Transient peak width change of the dysprosium peak as a function of the sample temperature.

Fig. 6 ▸ shows an example of XPP measurements on a heterostructure composed of a thin ∼10 nm Y capping layer, a 100 nm Dy layer followed by ∼5 nm Y and a ∼100 nm Nb buffer layer grown on a sapphire substrate by molecular beam epitaxy using the low-α mode of BESSY II. The complex heat transport phenomena in a similar sample with thicker Y layers was discussed by Koc *et al.* (2017*a* ▸) with an emphasis on the heat stored in the spin-system of Dy. Here we report on the sample with thinner Y capping layer and demonstrate how the increased time-resolution of the setup in low-α mode shown in Fig. 6 ▸(*a*) can quantify the acoustic oscillations of Dy in this heterostructure. The color map inset in Fig. 6 ▸(*a*) shows the simulation of the transient strain η (von Reppert *et al.*, 2020 ▸), which shows the complex spatio-temporal strain pattern in this heterostructure where the acoustic reflections at the substrate and at the sample surface dominate. The color of the inset reflects the strain: blue indicates compressive and red expansive strain, and *d* is the distance from the sample surface.

In Fig. 6 ▸(*b*) we show the transient strain of Dy for different sample temperatures. This illustrates the capabilities of the beamline regarding a variation of the sample temperature. In the antiferromagnetic state of Dy below its Neél temperature *T*_N_ = 179 K the material contracts upon photoexcitation. This contraction persists up to 100 ns – a timescale that can be addressed by the electronic variation of the pump–probe delay. The peak width change of the Dy Bragg reflection [Fig. 6 ▸(*c*)] encodes the inhomogeneous expansion of the Dy film: in the first few hundred picoseconds the energy density has a strong gradient over the first 100 nm metal film since the optical penetration depth of the pump pulse is only 22 nm. This steep exponentially decaying depth profile smears out slowly due to the slow heat transport in Dy (Koc *et al.*, 2017*a* ▸; von Reppert *et al.*, 2020 ▸). For the temperatures close to the phase transition, we observe a particularly interesting transient reduction of the peak width, which indicates that at the phase transition the spin fluctuations in the film support nanosize regions with smaller and larger lattice constant, depending on the local degree of spin disorder (Koc *et al.*, 2017*a* ▸). Laser-induced heating transiently brings the sample to a paramagnetic state which is characterized by a homogeneous crystal lattice. The inset in Fig. 6 ▸(*c*) shows a reciprocal space map to illustrate that we can separately analyze the out-of-plane or in-plane peak widths of the Bragg reflections.

### 3.2. Thermal transport: gold nanotriangles   

As an example of strongly mosaic samples, we now present measurements on triangular-shaped gold nanoparticles deposited on a Si substrate. The particles have a preferred (1 1 1) orientation with respect to their surface normal; however, there is a considerable mosaic spread of a few degrees because of the imperfect assembly of the triangles on the substrate. As thermal transport at interfaces is a technologically interesting topic of current fundamental research, such measurements of self-assembled particles are of great interest. Even small modification of interfaces due to roughness, structure, composition and/or homogeneity *etc*. can modify the transport properties significantly. We therefore studied the role of a chemical functionalization of Si substrates on the thermal conduction properties that are especially important on the nanoscale. Au nanotriangles (NTs) were synthesized and deposited on Si substrates (Liebig *et al.*, 2016 ▸, 2017 ▸) and the excitation of plasmons in such gold particles have been shown to drive chemical reactions via thermal pathways (Sarhan *et al.*, 2019*b* ▸).

**Figure 7:**
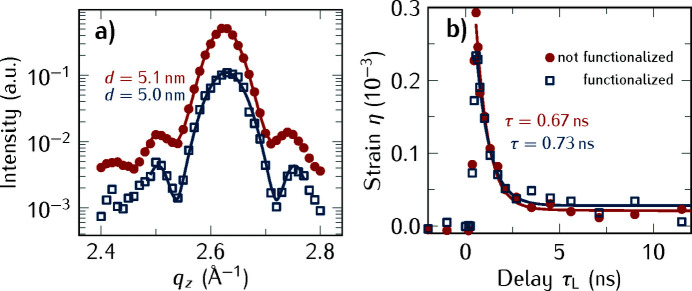
(*a*) ω scan of the 111 Bragg reflection of Au nanotriangles on a bare Si substrate (filled blue symbols) and a Si substrate that has been functionalized prior to the NT deposition (open red symbols). In both cases, the Laue oscillations yield a thickness of the Au NTs of ∼5 nm. (*b*) Temporal evolution of the strain in the Au NTs after laser excitation with λ = 1028 nm and *F* = 0.1 mJ cm^−2^.

In the following, we present the comparison of two samples, one where the NTs were deposited on an as-received Si substrate and the other one where the NTs have been deposited with a comparable coverage on a 3-methacryloxy­propyltrimethoxysilane (3-MTPS) functionalized Si substrate. For the first sample, the Si substrate was only cleaned before the Au NTs were deposited. For the second sample, the substrate was additionally functionalized with 3-MPTS molecules before the NTs were transferred onto it (Liebig *et al.*, 2017 ▸). Prior to the optical excitation of the Au NTs with laser pulses of λ = 1028 nm, RSM scans of the samples were performed around the Au 111 Bragg reflection [Fig. 7 ▸(*a*)], which indicates an interplanar distance of 0.239 nm of the Au 111 planes corresponding to a lattice constant of the Au NTs of 0.414 nm. The Laue oscillations around the 111 Bragg reflection of Au imply a coherence length of the NTs of ∼5 nm, a value slightly smaller than the layer thickness retrieved from X-ray reflectivity and femtosecond X-ray diffraction measurements on similar samples (von Reppert *et al.*, 2016*b* ▸; Liebig *et al.*, 2017 ▸; Sarhan *et al.*, 2019*a* ▸).

In Fig. 7 ▸(*b*) we show the transient strain in the Au NTs after laser excitation with the fluence *F* = 0.1 mJ cm^−2^. We compare the heat flow into the functionalized and not-functionalized substrates. The solid lines in Fig. 7 ▸(*b*) are an exponential fit to the experimental data and we find that the functionalized substrate has a ∼10% slower decay time, hence its thermal conduction is only reduced marginally. This is in line with time-domain thermoreflectance experiments on self-assembled monolayers of ∼1 nm-long molecules connecting Au and Si, where the interface conductance for molecules depends on the thiol bond (Losego *et al.*, 2012 ▸). Thus, we attribute the reduction of the thermal conduction to the marginally increased thermal interface resistance caused by the 3-MPTS functionalization of the Si surface. The functionalization is likely accompanied by an increased distance between the Au NTs and the Si substrate, reducing the thermal conductivity (von Reppert *et al.*, 2016*b* ▸).

### 3.3. Operando studies of ferroelectric devices   

**Figure 8:**
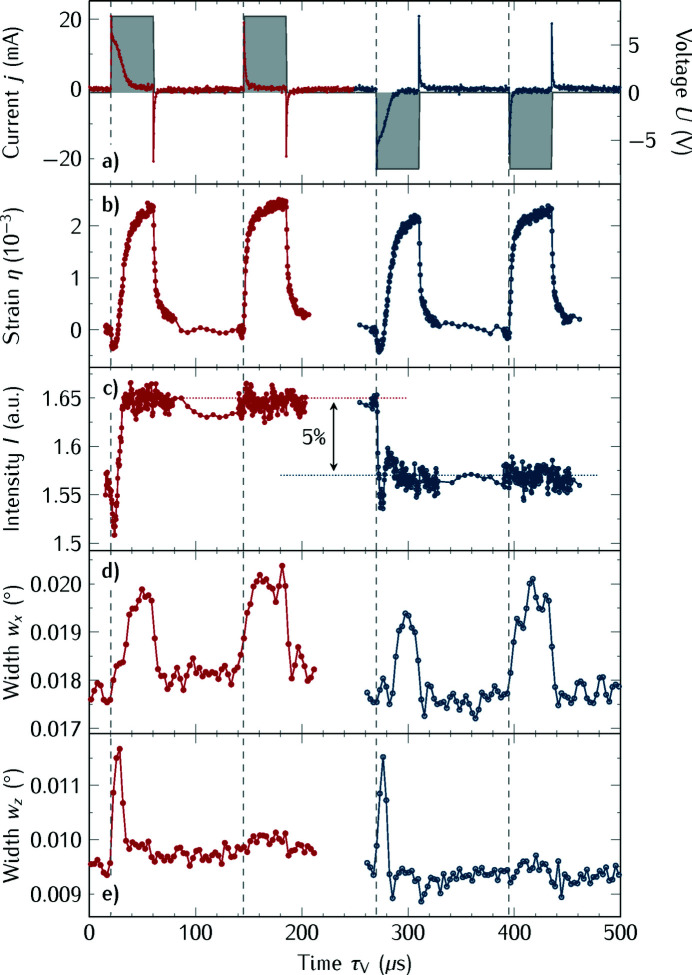
Simultaneously measured electrical and structural response of a PZT thin film as a function of the delay between onset of the electric field and the X-ray probe, τ_V_. In (*a*) we show the switching current and overlaid with gray shading the applied PUND voltage sequence that indicates when the field is applied to the sample. The strain η obtained from the 002 Bragg reflection is shown in (*b*) and the intensity of the Bragg reflection is shown in (*c*). The width as function of the delay τ_V_ is plotted in (*d*) and (*e*) for the in-plane and out-of-plane components, respectively. The dashed gray vertical lines indicate the beginning of the PUND pulses.

Last we present the dynamics of typical FE switching experiments where the dynamics of the sample are triggered by electrical field pulses as opposed to light pulses. We show the measurement of the ferroelectric polarization reversal, which is an important topic, especially for the development of new, fast persistent data storage devices. For most studies of the structure dynamics in ferroelectrics the accompanying electronic response must be measured separately (Grigoriev *et al.*, 2008 ▸, 2009 ▸, 2011 ▸). Now, at the KMC-3 XPP beamline it is possible to measure simultaneously the structural and electrical response of working ferroelectric devices. In contrast to setups for powder diffraction (Ryding *et al.*, 2013 ▸), which can also be extended to thin films (Wooldridge *et al.*, 2012 ▸), our setup is optimized for the investigation of crystalline thin film samples. We demonstrated for crystalline Pb(Zr_0.8_Ti_0.2_)O_3_ (PZT) thin films, which were grown by pulsed laser deposition on a SrRuO_3_ bottom electrode, that the depolarization field plays an important role during the FE polarization reversal (Kwamen *et al.*, 2017 ▸). In Fig. 8 ▸ we show exemplarily the electrical response of a 200 nm-thick PZT sample.

θ–2θ scans were performed at a repetition rate of the PUND sequence of 2 kHz. Thus, the 500 µs-long pulse period was divided into several 33 µs-long intervals that were covered by the PicoHarp acquisition window and stacked together. This in turn means that the time resolution per channel of 512 ps is still much shorter than the rise time of the scintillator material and, hence, the measured data were binned to yield an effective time resolution of 2 ns.

In Fig. 8 ▸(*a*) we display the measured switching current, *j*, together with the applied PUND voltage sequence: the gray area indicates the time when the field is applied with amplitude *U* = ±7.5 V, far above the coercive field of *U*_c_ = 4 V for this contact. The vertical dashed lines in Fig. 8 ▸ indicate the onset of the voltage pulses. The simultaneously measured structural response of the 002 Bragg reflection of PZT, the strain η(τ_V_) = *c*(τ_V_)/*c*(0) − 1, is shown in Fig. 8 ▸(*b*). The integrated peak intensity *I*(τ_V_), which is proportional to the unit cell structure factor (Gorfman *et al.*, 2013 ▸), is plotted in Fig. 8 ▸(*c*) for both applied polarities. The intensity difference of 5% clearly indicates the FE polarization reversal of the PZT layer (Gorfman *et al.*, 2016 ▸; Kwamen *et al.*, 2017 ▸). The additional decrease of the peak intensity right after the onset of the polarization switching voltage pulse within 10 µs was ascribed to additional disorder of the Ti central cation and the surrounding O octahedra (Kwamen *et al.*, 2017 ▸). These measurements were made using the fast scintillator with the photomultiplier tube and a vertical slit in front of the detector that limits the acceptance of X-rays scattered with *q*_*x*_ ≠ 0. They are consistent with data obtained using the gated area detector for the analysis of the time-resolved peak width change along *q*_*z*_, *w*_*z*_, of the PZT Bragg reflection. In order to separate the in-plane and out-of-plane peak width contributions [Figs. 8(*d*) and 8(*e*) ▸] we used the gated pixel detector, which can only use the photons in the camshaft bunch, therefore such full RSM measurements take longer: the measurement of one RSM at each delay τ_V_ took 5–6 min under these experimental conditions, that is, it is possible to measure 120 delay steps in 12 h. For comparison, the full sampling of the PUND sequence of the same 200 nm-thick PZT film with the PMT requires less than 8 h at the repetition rate of 2 kHz. At higher repetition rates, in particular faster than 33 kHz, the PicoHarp time trace covers the complete pulse sequence, which speeds up detecting the full sequence and only one θ–2θ scan is sufficient. On the other hand, the area detector allows us to reconstruct the RSM around the Bragg reflection and thus it becomes possible to separate the symmetrically and asymmetrically scattered X-rays, that is, to distinguish the peak width changes for the out-of-plane and in-plane directions. These measurements clarify that the in-plane and out-of plane peak widths obviously measure significantly different dynamics: only the in-plane peak width is sensitive to the domain *motion* during the switching at about 20 and 270 µs.

## 4. Summary and outlook   

In this paper we presented a comprehensive overview of the possibilities at the KMC-3 XPP endstation installed at the synchrotron radiation facility BESSY II. This hard X-ray diffraction beamline is designed for pump–probe measurements of the structural response of bulk samples, thin films or heterostructures after optical and/or electrical excitation with a time-resolution of 80 ps in hybrid mode and about 17 ps in the low-α mode, the latter being usually offered two weeks per year. The upgraded laser setup for optical excitation allows for different excitation schemes (wavelength, repetition rate, pulse energy) and can flexibly be extended. For example we have implemented a multipulse setup for generating pulse sequences that can be used to excite monochromatic phonon wavepackets (Bojahr *et al.*, 2013 ▸). A new non-collinear optical parametric amplifier will allow a more specific excitation of samples in the optical spectral region. In addition, it is possible to integrate lasers brought by users to the beamline into the experimental setup within the laser hutch and use them for particular tasks, for example for a specific sample heating (Sarhan *et al.*, 2019*a* ▸).

Thermal transport in layered nanostructures as well as in ordered nanoparticle layers is a topic that has attracted a lot of attention in the last years. The KMC-3 XPP endstation is capable of investigating the heat flow in such samples, which proceeds on sub-nanosecond to microsecond timescales. The electronic delay between optical excitation pulse and X-ray probe pulse facilitates such investigations on multiple timescales. Hard X-ray sensing of thermal transport in buried detection layers within the sample exploit the material-selectivity and deep penetration depth of hard X-rays. This was demonstrated on the rare earth magnet Dy where the magnetic subsystem plays a crucial role for the thermal heat transport, especially in the antiferromagnetic phase. Another example was the modified thermal transport in Au nanotriangles deposited on Si. The addition of linker molecules between the Si substrate and the nanotriangles modifies the heat transport in such nanostructures by 10%.

For lower time resolution of 2 ns fast detectors are used to analyze for example the polarization reversal of ferroelectric thin films. The structural response can be simultaneously measured with the electrical response of a working device. This has shed new light on the role of the depolarization field (Kwamen *et al.*, 2017 ▸) and the domain wall dynamics (Kwamen *et al.*, 2019 ▸), which is particularly important for the understanding of the dynamics of the switching process of ferroelectric devices. We have recently combined the two scenarios, that is, modified the electrically induced ferroelectric switching by simultaneous laser-irradiation of the device. Such studies hold great future promise for overriding the speed limit of ferroelectric switching.

## Figures and Tables

**Table 1:** Photon flux for different photon energies at the KMC-3 beamline at the X-ray focus position as obtained with an evacuated sample chamber during hybrid mode operation, single bunch operation, and low-α operation, respectively

Energy (keV)	5	8	10
Hybrid mode			
Flux (photons s^−1^)	2 × 10^11^	1 × 10^11^	2 × 10^10^
Effective flux when selecting the single bunch at a 1.25 MHz repetition rate (gated detector)	3 × 10^9^	2 × 10^9^	3 × 10^8^

Single bunch mode			
Effective flux when selecting the single bunch at a 1.25 MHz repetition rate (gated detector)	1 × 10^10^	5 × 10^9^	1 × 10^9^

Low-α mode			
Flux (photons s^−1^)	6 × 10^10^	3 × 10^10^	6 × 10^9^
Effective flux when selecting the single bunch at a 1.25 MHz repetition rate (gated detector)	2 × 10^8^	1 × 10^8^	2 × 10^7^
